# Arterial compliance in a group of normotensive and untreated hypertensive Cameroonian subjects in Yaounde

**DOI:** 10.11604/pamj.2016.24.162.7526

**Published:** 2016-06-27

**Authors:** Samuel Kingue, Joshua Walinjom, Alain Menanga, Pierre Mintom, Marie Ntep Ngweth, Fesuh Betrand, Walinjom Muna

**Affiliations:** 1 Medical & cardiology Unit, General Hospital Yaoundé, Cameroon; 2 Faculty of Medicine and Biomedical sciences, University of Yaoundé I, Cameroon; 3 Medical Unit, Central Hospital Yaoundé, Cameroon; 4 National Advanced School of Engineering, Department of mathematics, Physics and Applied Statistics, University of Yaoundé 1, Cameroon

**Keywords:** Arterial compliance, pulse-wave velocity, hypertension, Black African

## Abstract

**Introduction:**

Arterial compliance is an independent predictor of cardiovascular events. It decreases with age and this decrease is accelerated by hypertension. The objectives were to determine the arterial compliance in a group of normotensive and untreated hypertensive stage 1, 2 and 3 Cameroonian subjects.

**Methods:**

A cross-sectional study was conducted from August 2012 to February 2013 in Yaoundé. Our sample size was 88 participants. The PulsePen^®^ device was used to determine cfPWV (carotid-femoral Pulse Wave Velocity) and central Augmentation Index % (AIx). Other measurements obtained were: blood pressure (BP), body mass index (BMI), fasting glycaemia, lipid profile and serum creatinine.

**Results:**

Our sample's mean age was 35.48 years and ranged from 20 to 60 years. The means of: cfPWV, SBP, DBP, Pulse Pressure (PP) and Heart Rate (HR) showed a statistically significant increase (p-value < 0.05) across the groups from normotensive to severely hypertensive patients. cfPWV was significantly correlated (p-value< 0.05) to: Age, Central SBP, Central DBP, Central PP, HR, BMI and central Augmentation index (AIx). Furthermore, cfPWV was significantly dependent on LVH (p-value <0.05).

**Conclusion:**

This study suggests that arterial compliance decreases with increase severity of hypertension, indicating a higher risk of developing cardiovascular events in severely hypertensive patients.

## Introduction

Arterial compliance decreases with age and this decrease is accelerated by hypertension. Carotid-femoral pulse wave velocity (PWV), the current 'gold-standard' measure of arterial compliance, has emerged as an important independent predictor of cardiovascular events in hypertensive patients [1]. Arterial stiffness is associated with an increase in SBP and PP, raising left ventricular afterload and myocardial work, leading to hypertrophy with reduced coronary perfusion, which may result in sub-endocardial ischemia [2]. The European Society of Hypertension (ESH) and European Society of Cardiology (ESC) published guidelines declaring a borderline or threshold value of aortic PWV (>12 m/sec) that should be used to stratify cardiovascular risks in hypertensive patients [3]. To the best of our knowledge, no study has been done in sub Saharan Africa to determine the arterial compliance in hypertensive subjects. We therefore sought to determine the arterial compliance in a group of normotensive and untreated hypertensive stage 1, 2 and 3 subjects in Yaounde.

## Methods

In a cross-sectional study, we recruited 103 Cameroonians subjects through free hypertension screening campaigns organized in public places. Participants were also recruited amongst patients consulting at the cardiology outpatient clinic of the Yaoundé General and Central Hospitals from August 2012 to February 2013. Included in our study were consenting Normotensive and untreated hypertensive subjects aged from 20 to 60 years. We excluded: patients with Diabetes, subjects with a renal disease, pregnant women, subjects in an active infectious state and patients with incomplete data. During clinical clerking, blood pressure, temperature, weight and height were recorded. We also did a fasting glycaemia and a urine Beta Human Chorionic Gonadotropin pregnancy test was done in women suspected to be pregnant. Blood samples were later collected in test tubes and taken to the laboratory to analyze serum creatinine and lipid profile. Of the 103 recruited subjects, 15 were excluded as follows: 05 participants had a positive pregnancy test (During normal pregnancy, arterial stiffness has been shown to increases from the midtrimester to term) [4], 03 had a fasting glycaemia > 1.26g/l (diabetes is associated with increase arterial stiffness) [5], 04 had serum creatinine >1.6 mg/dl (Reduced arterial elasticity has been shown in patients with renal impairment) [6] and 03 presented with incomplete data. Our final sample size involved a total of 88 participants 25 of whom were normotensive while 22, 21 and 20 were untreated hypertensive stages 1, 2 and 3 subjects respectively as per ESC/ESH 2007 Classification of hypertension.

### Arterial stiffness Measurement

#### Pulse wave velocity (PWV)

**Figure 1:**
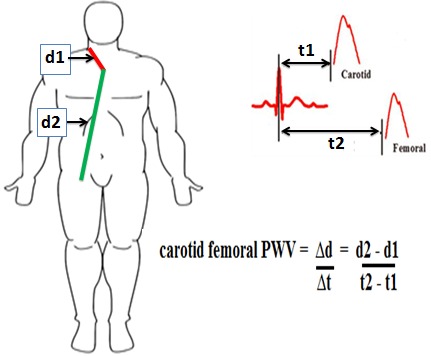
Measurement of PWV

PWV is the 'gold-standard' measurement of arterial stiffness. It was determined by means of a PulsePen^®^ device (Dia- Tecnesrl, Milan, Italy), a non-invasive validated, easy to use and high-fidelity tonometer. Briefly, the PulsePen is comprised of one tonometer and an integrated ECG unit. Both pressure and electrocardiographic signals are transmitted to a computer by means of an optical fiber. Central artery pressure waveforms were evaluated noninvasively using applanation tonometry [7, 8]. All measurements were performed in a quiet room by the same operator who was specially trained. The subject was asked to lie down for 10-15minuite in the supine position after which his blood pressure was recorded using an electronic sphygmomanometer (HEM-OMRON^®^705, Omron Corporation, Tokyo Japan). The subject's identification, age, height, weight and blood pressure were entered into the pulsepen software. After placing the ECG electrodes, the common carotid artery pulse was palpated and the waveform recorded by gently placing the tonometer at a right angle to the artery. ECG was recorded simultaneously. Then the femoral artery was palpated followed by waveform recording. ECG was also recorded simultaneously. The blood pressure was measured before and after waveform recordings. A measuring tape was used to get the distance from: the supra-sternal notch to the carotid point of tonometer application (d1), the supra-sternal notch to the femoral point of tonometer application (d2). The difference between these distances (d2-d1) was then entered manually into the Pulsepen software. The pulsepen software automatically determined the pulse wave delay time by calculating the time elapsed from the peak of the R wave to the 'foot' of the pressure pulse contour. The device automatically measured the pulse wave delay time from the: R wave of the ECG qRs complex to the 'foot' of the carotid pressure pulse contour (t1), R wave of the ECG qRs complex to the 'foot' of the femoral pressure pulse contour (t2). Carotid-femoral PWV was defined as, the difference in distance (Δd) of the pulse wave transit which represents the difference between the distance from the supra-sternal notch to the femoral point of tonometer application (d2) and the distance from the supra-sternal notch to the carotid point of tonometer application (d1) divided by the time difference (Δt) between the time delay from the R wave of the ECG qRs complex to the 'foot' of the femoral pressure pulse contour (t2) and the delay time from the R wave of the ECG qRs complex to the 'foot' of the carotid pressure pulse contour (t1). This is shown on Figure 1. **(Carotid-Femoral PWV = Δd/Δt= d2-d1/t2-t1).**

#### The augmentation index (AIx)

Augmentation index(AIx) is an important arterial stiffness parameter. It was measured by automatic identification of the “first shoulder” (inflection point) on the average carotid pulse signal by the pulsepen software. AIx= 100 x (Augmentation pressure / pulse pressure). To assure that alterations in blood pressure and heart rate did not bias the results of stiffness assessment, the Pulsepen software automatically rejected measurements in which blood pressure or heart rate changed by more than 5% during the time between the sequential carotid and femoral pulse wave recordings. For reliable results, only high-quality recordings were included in our analysis defined as an in-device quality index of >80%. Carotid-femoral or aortic PWV (cfPWV), Central SBP mmHg, Central DBP mmHg, Central PP mmHg, Heart rate beats/min, MAP and central Augmentation index % (AIx) are arterial stiffness parameters whose values were given automatically by the device. Only measurements meeting Pulsepen^®^ quality control criteria were accepted.

### Data analysis

Data was analyzed using the R-software and Microsoft Excel. Univariate Analysis was used to determine the mean, SD and range of quantitative variables. Pearson's chi square test was used to show the independence of qualitative variables and Student's t-test for equality of means in the case where the distribution of the variables were normal. Otherwise the Mann-Whitney test was used. The Kruskal-Wallis rank sum test was used to test for independence between qualitative and quantitative variables. We used the Kendall's tau rank test to test for correlation between quantitative variables in the case where the distribution was not normal. Jonckheere's trend test was used to test for the trend of means of quantitative variables across the groups. Statistical significance was set at the 5% interval.

## Results

**Figure 2:**
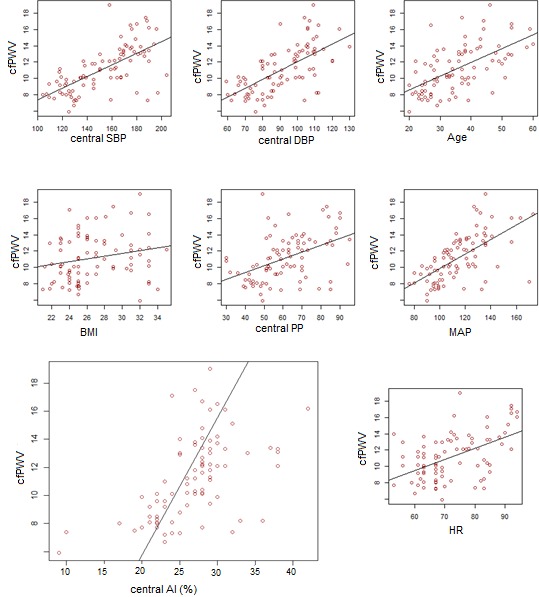
The relationship between cfPWV and: central SBP, central DBP, Age, BMI, central PP, MAP, Central AIx and HR

Of the 88 participants studied, 51.1% were females and 48.9% were males. The mean age of the study population was 35.48 years and ranged from 20 to 60 years. The mean of hemodynamic parameters (cfPWV, Central SBP, central DBP, Central PP, MAP, HR, and Central Augmentation Index (AIx) ) showed a statistically significant increase (p-value < 0.05) as we moved across the groups from normotensive to severely hypertensive patients as seen on Table 1. Although there was a statistically significant increase (p value < 0.05) in the mean age across the groups, fasting blood sugar (FBS), high density lipoprotein cholesterol (HDL-C), total cholesterol (TC) and triglycerides (TG) showed no statistically significant increase (p-value > 0.05) in their means across the different groups as seen on Table 2. We can see from Figure 2 that, cfPWV was significantly correlated to Age (r=0.4, p<0.001), Central SBP (r=0.51, p<0.001), Central DBP (r=0.47, p<0.001), Central PP (r=0.34, p<0.001), MAP (r=0.5, p<0.001), HR (r=0.34, p<0.001), BMI (r=0.16, p<0.027), and central AIx(r=0.45, p<0.001). On the other hand, cfPWV was not significantly correlated (p-value >0.05) to TG, TC and FBS. Also, cfPWV was significantly dependent on LVH (p-value <0.05).

**Table 1:** Hemodynamic parameters in normotensive and hypertension stage1,2,3 groups

Variable	Normotensive	Stage 1	Stage 2	Stage 3	p-value
**cfPWV [m/s]**	7.08 ± 1.28	8.01 ± 1.37	10.09 ± 1.73	12.84 ± 2.17	<0.001
**Central SBP [mmHg]**	120.56 ± 7.75	143.75 ± 10.55	169.90 ± 11.04	181.86 ± 10.44	<0.001
**Central DBP [mmHg]**	72.96 ± 8.03	86.85 ± 8.19	103.05 ± 13.07	106.77 ± 7.04	<0.001
**Central PP [mmHg]**	47.20 ± 9.85	57.35 ± 9.80	66.05 ± 10.93	76.27 ± 13.32	<0.001
**MAP [mmHg]**	92.68 ± 6.82	106.70 ± 5.03	121.14 ± 6.42	140.27 ± 14.63	<0.001
**HR [beats/min]**	67.40 ± 7.88	68.60 ± 7.32	71.57 ± 10.36	82.91 ± .27	<0.001
**Central.Aug.Index [AIx]%**	18 ± 11.4	20 ± 10.3	26 ± 9.7	29 ± 12.3	<0.001

**Table 2:** Clinical and paraclinical characteristics in normotensive and hypertension stage 1, 2 and 3 groups

Variable	Normotensive	Stage 1	Stage 2	Stage 3	p-value
**Age[years]**	25.64 ±3.64	34.95 ± 6.50	38.24 ±7.53	44.50 ± 9.75	<0.001
**BMI [kg/m^2^]**	26.86 ± 3.95	26.14 ± 3.05	26.79 ± 3.87	27.06 ± 3.60	0.543
**FBS[g/dl]**	0.85 ±0.11	0.91 ±0.14	0.97 ± 0.15	0.82 ± 0.12	0.949
**HDL.Cholesterol [g/l]**	0.73 ± 0.15	0.70 ±0.14	0.72 ± 0.13	0.76 ±0.13	0.469
**Total Cholesterol [g/l]**	1.97 ± 0.39	2.04 ± 0.40	2.09 ±0.39	2.74 ± 3.21	0.190
**Fasting.Triglyceride [g/l]**	0.96 ± 0.44	1.13 ± 0.39	1.12 ± 0.44	1.04 ± 0.41	0.395

## Discussion

The main objective of this study was to determine the arterial compliance in a group of normotensive and untreated hypertensive stage 1, 2 and 3 subjects in a sub-Saharan African setting. Untreated patients were studied in order to avoid the effect of some anti-hypertensive drugs like calcium channel blockers and ACE inhibitors which may ameliorate arterial compliance [9].

### Relationship between cfPWV and BP parameters

CfPWV was positively correlated to BP parameter (SBP, DBP, PP and MAP). Ngim et al [10] in 1999, reported that carotid-femoral PWV was positively correlated with SBP and MAP in untreated hypertensive and normotensive middle aged Malay men. Stompor et al [11] in 2003 in Poland also found out that aortic PWV was correlated significantly with SBP, MAP and PP. Yasmin et al [12] in 1999 in the United Kingdom found out that, PWV correlated with both SBP and DBP in 105 offspring of patients with familial hypertension. Franklin et al [13] in 1999 in California reported a positive association between cardiovascular mortality and DBP before 60 years of age and a negative association thereafter. They as well stated that there is epidemiological evidence that SBP and DBP increase markedly with age. However, after the age of 50 to 60 years, the rise in DBP tends to disappear, and DBP may even decrease with age. Thus, PP increases more markedly with age than does MBP. This may explain why cfPWV was correlation with DBP in our study as we had a young study population. Gradual progression of atherosclerosis of the abdominal aorta occurs with increasing levels of systolic blood pressure [14], suggesting that systolic blood pressure is related to the atherosclerotic process and arterial stiffness. Our study showed a gradual increase in PWV with rising levels of systolic blood pressure which is in accordance with these findings. These data may indicate that a reduced arterial compliance may lead to an elevation of systolic blood pressure. Blood pressure elevation sets a pathophysiological process in progress. Atherosclerosis may lead to a further reduction of the compliance of the large arteries and a subsequent rise in systolic blood pressure [14]. This process may enhance arterial stiffness and atherosclerosis, leading to a further increase in systolic blood pressure and to development of hypertension. Additionally, hypertension could further enhance the development of atherosclerosis. Therefore, severe hypertensive patients may present with atherosclerosis and decreased arterial compliance especially with advancing age as it was the case in our study. Wallace et al [15] in 2007 in the United Kingdom showed that patients with isolated systolic hypertension have higher aortic PWV and decreased endothelial function compared with age-matched control subjects. Endothelial micro particles (EMP) and Platelet microparticle (PMP) which are markers for endothelial injury/activation contribute to the pathogenesis of vascular injury observed in patients with uncontrolled severe hypertension. Preston et al [16] in 2003 in Miami showed that EMP and PMP are significantly elevated in high-risk patients with severe hypertension. This finding is consistent with the hypothesis that endothelial and platelet activation may play a role in the pathogenesis of the accelerated target organ injury observed in patients with severe uncontrolled hypertension. EMP and PMP have diverse effects on coagulation, leukocytes, platelets, and endothelium that could ultimately contribute to the pathogenesis of the acute vascular injury observed in patients with uncontrolled severe hypertension. This can therefore explain arterial stiffness in patients with severe hypertension bringing out the reason for the progressive increase in the value of arterial stiffness parameters across normotensive, and hypertension stage 1, 2 and 3 patients.

### Relationship between cfPWV and age

In our study, PWV was positively correlated to age. Avolio et al [17] showed that, Arterial stiffness increases with age by approximately 0.1 m/s/y in East Asian populations with low prevalence of atherosclerosis. The progressive increase in age as we move from normotensive to hypertension stage 3 patients may explain the progressive increase in PWV with severely hypertensive patients having the highest PWV values. Medial degeneration, a consequence of aging, appears to be the most important factor. Medial calcification develops with age leading to reduced elasticity and arterial stiffness [18]. In the intima, predominant changes include increase in intima media thickness and prevalence of atherosclerosis. Age-related increase in PWV is found in populations with low prevalence of atherosclerosis, suggesting that medial degeneration is an important cause of arterial stiffening [19]. One hypothesis is that repetitive cyclic stress over a life span is responsible for the fracture of elastin fibres. This suggests that stiffness is an inevitable consequence of ageing. An understanding of molecular pathways that influence arterial stiffness is now emerging. Ageing of the arterial media is associated with increased expression of matrix metalloproteinases (MMP), which are members of the zinc-dependent endopeptidase family and are involved in degradation of vascular elastin and collagen fibres. With advancing age, structural changes of collagen fibres, in particular collagen cross-linking by advanced glycationendproducts (AGEs), may also lead to arterial stiffness [19].

### Relationship between cfPWV and HR

In this study we found a statistically significant link between increased rigidity of central arteries and an elevated heart rate, a major risk factor of cardiovascular diseases. In an observational study, Sa Cunha et al [20] in 1997, showed that high heart rate was strongly associated with elevated PWV in a population of normotensive and hypertensive subjects in Paris. One of the basic concepts used to understand the relationships between heart rate and arterial stiffness is the stress-strain relationship of blood vessels. Under dynamic conditions, this relationship is highly frequency-dependent, because of the viscous properties of the arterial wall. In fact, because the viscous component of the elastic artery is highly known to be velocity dependent, it is expected that higher values of heart rate, might be associated with more reduced distensibility. Thus for a given value of blood pressure, arterial stiffness is expected to be higher when the heart rate is increased. The increase in heart rate is associated with reduced distension capacities of the carotid artery [21]. Coronary atherosclerosis in humans has been shown to be strongly and independently associated with an increased heart rate and increased aortic stiffness [22].

### The relationship between cfPWV and central augmentation index

In our study, cfPWV was positively correlated to central Augmentation. Yasmin and Brown [12] in 1999 in the United Kingdom showed that, PWV was strongly correlated with augmentation index in 105 offspring of patients with familial hypertension. Augmentation index is a sensitive marker of arterial status. It is known to be a predictor of adverse cardiovascular events in a variety of patient populations, and higher augmentation index is associated with target organ damage.

### The relationship between arterial stiffness and LVH

cfPWV was significantly dependent on LVH. Darne et al in 1989 [23] and Girerd et al [24], showed that Arterial stiffness is associated with left ventricular hypertrophy in normotensive and hypertensive patients respectively. Arterial stiffness is a cause of premature return of reflected waves in late systole, increasing central pulse pressure and the load on the ventricle which reduces ejection fraction and increases myocardial oxygen demand. Left ventricular hypertrophy is a known risk factor for congestive heart failure and cardiovascular events. The elevation of SBP, which raises left ventricular afterload and myocardial work, reduces coronary perfusion, result in subendocardial ischemia [25]. LVH is a known marker of cardiovascular events and therefore indicates a higher risk of cardiovascular events in hypertensive patients.

### Clinical, laboratory and hemodynamic characteristics in normotensive and hypertension stage 1, 2, 3 groups

Looking at the means in the normotensive, hypertension stage 1, 2 and 3 groups, There was a statistically significant increase in the mean Age and mean of all hemodynamic parameters (PWV, SBP, DBP, PP, HR, central AIx and MBP) across the groups with increase severity of hypertension. The decrease in arterial compliance across the groups with increasing hypertension stage may be due to the fact that, increase in blood pressure causes endothelial injury leading to impaired nitric oxide availability which has a vital role in vasodilation thereby favouring increase arterial stiffness. On the other hand; BMI, FBS, HDL-C, TC and TG showed no statistically significant increase in their means across the different groups.

### cfPWV relationship with cholesterol, BMI and FBS

cfPWV was not significantly related to total cholesterol, triglycerides and HDL-C. Our results were similar to those of EJ Kim et al [26] who in 2007 found that PWV was not significantly related to total cholesterol, triglycerides, HDL-C and fasting glucose in normotensive and hypertensive Korean subjects. Amar et al [27] in France in 1997 had similar results. Our findings also showed that PWV was significantly related to BMI. This is similar to results by Ronnback et al [28] in 2007 who showed that PWV was correlated to BMI in middle-aged Finland men.

## Conclusion

This study suggests that arterial compliance decreases with increase severity of hypertension, indicating a higher risk of developing cardiovascular events in severely hypertensive patients.

### What is known about this topic

- Arterial compliance decreases with age and this decrease is accelerated by hypertension;
- Arterial Compliance is an independent predictor of cardiovascular Events;
- Arterial stiffness is associated with an increase in Systolic Blood Pressure and Pulse Pressure, raising left ventricular afterload and myocardial work, leading to hypertrophy with reduced coronary perfusion, which may result in sub-endocardial ischemia.

### What this study add

- Arterial Compliance in hypertensive patients in a typical Sub-Saharan African setting;
- Arterial compliance in different stages of hypertension;
- Arterial compliance in Sub-Saharan African patients with left ventricular hypertrophy.
